# Lack of an Antibacterial Response Defect in *Drosophila Toll-9* Mutant

**DOI:** 10.1371/journal.pone.0017470

**Published:** 2011-02-28

**Authors:** Karine Narbonne-Reveau, Bernard Charroux, Julien Royet

**Affiliations:** Institut de Biologie du Développement de Marseille-Luminy (IBDML), CNRS UMR 6216/Aix-Marseille II University, Campus de Luminy, Marseille, France; University of Texas MD Anderson Cancer Center, United States of America

## Abstract

Toll and Toll-like receptors represent families of receptors involved in mediating innate immunity response in insects and mammals. Although *Drosophila* proteome contains multiple Toll paralogs, Toll-1 is, so far, the only receptor to which an immune role has been attributed. In contrast, every single mammalian TLR is a key membrane receptor upstream of the vertebrate immune signaling cascades. The prevailing view is that TLR-mediated immunity is ancient. Structural analysis reveals that *Drosophila* Toll-9 is the most closely related to vertebrate TLRs and utilizes similar signaling components as Toll-1. This suggests that Toll-9 could be an ancestor of TLR-like receptors and could have immune function. Consistently, it has been reported that over-expression of Toll-9 in immune tissues is sufficient to induce the expression of some antimicrobial peptides in flies. These results have led to the idea that Toll-9 could be a constitutively active receptor that maintain significant levels of antimicrobial molecules and therefore provide constant basal protection against micro-organisms. To test theses hypotheses, we generated and analyzed phenotypes associated with a complete loss-of-function allele of Toll-9. Our results suggest that Toll-9 is neither required to maintain a basal anti-microbial response nor to mount an efficient immune response to bacterial infection.

## Introduction

Innate immunity is a rapid and efficient response that multicellular organisms mount to defend themselves against infection and that has been conserved throughout evolution (reviewed in [1], [2]). Upon infection, microbe-specific immune elicitors, known as microbe-associated molecular patterns (MAMPs), are recognized by a set of so-called pathogen recognition receptors (PRRs) [3], [4]. Mainly through genetic screens, the *Drosophila* community has largely contributed to the molecular dissection of the signaling modules that regulate expression of immune genes following infection. *Drosophila* humoral immune response is under the transcriptional control of two NF-κB signaling cascades, the Immune deficiency (Imd) and the Toll pathways, which closely resemble mammalian TNF-R and Toll-Like Receptor (TLR), respectively (reviewed in [5], [6]). The Imd pathway can be activated by Gram-negative bacteria that contain diaminopimelic acid type peptidoglycan (DAP-type PGN) in their cell wall. Binding of DAP-type PGN to the transmembrane receptor Peptidoglycan-Recognition-Receptor-LC (PGRP-LC) induces phosphorylation and proteolytic activation of the NF-κB/Rel transcription factor Relish. This, in turns, activates the expression of several antimicrobial peptides (AMPs), such as Diptericin [2], [7], [8]. The Toll pathway is triggered by Gram-positive bacteria with Lysine-type peptidoglycan (Lys-type PGN), fungi and yeast, and mediates phosphorylation and subsequent degradation of the IκB-like inhibitor Cactus, freeing the NF-κB transcription factor Dif that translocates into the nuclear compartment where it induces the expression of several genes encoding AMPs, including *Drosomycin* and *Cecropin* [9]–[13].

The *Toll* (also known as Toll-1) gene was originally isolated for its role in specifying dorso-ventral polarity of the *Drosophila* embryo [14]. Toll encodes a type I transmembrane receptor composed of three domains: the extracellular leucine-rich repeats (LRR) domain, containing tandem arrays of a short leucine-rich motif in the N-terminal region, a sequence likely to form a single transmembrane helix, and an intracellular C-terminal domain significantly related to the vertebrate interleukin-1 receptor (the Toll-interleukin receptor -TIR- domain), involved in signaling processes [15]–[17]. Although it is well established that vertebrates TLRs function as PRRs for numerous microbial ligands such as LPS, dsRNA, Flagellin, DNA CpG (reviewed in [18], [19]), there are so far no indication that *Drosophila* Toll family members are able to directly recognized microbial motifs. How then is the Toll receptor activated by bacterial PGN in flies? Recognition of bacterial PGN by PGRP-SA triggers a proteolytic cascade whose last substrate is the circulating pro-cytokine Pro-Spätzle [20], [21]. This proteolytic cleavage transforms the immature cytokine Spätzle into an active ligand which triggers Toll receptor dimerization [22]–[24]. Receptor dimerization allows the recruitment of an adaptator complex containing three intracellular Death domain-containing proteins, MyD88, Tube and Pelle [12], [25], [26]. Then, by a still unknown mechanism, Cactus is phosphorylated, allowing the release of the Dif transcription factor [27], [28].

In addition to the family founder Toll-1, the *Drosophila* genome contains 8 additional members: Toll-2 also known as 18-Wheeler, and Toll-3 to Toll-9 [29], [30], the function of some of which have been recently studied. Toll-8, also known as Tollo, is required for the induction of neural specific glycosylation in the *Drosophila* embryo [31], [32]. Initially thought to be the membrane receptor upstream of the Imd cascade, Toll-2/18-W is now believed to function in the embryonic epithelium by regulating cell apical constriction via the Rho-GTPase-signaling pathway [30]. More recently, by analyzing double loss-of-function mutants, Yagi et al. showed that 18-W, Toll-7 and Toll-8 may have redundant functions in regulating developmental processes [33]. Although limited to some of the Toll-related proteins, these studies tend to indicate that the implication of Toll family members in immunity is rather the exception (Toll-1) than the rule.

Phylogenetic analysis of TIR domains indicate that, with the exception of Toll-9, *Drosophila* Tolls are more closely related to each other than to mammalian TLRs [34]. This suggests that these two groups of receptors, *Drosophila* Tolls and mammalian TLRs, evolved independently to carry distinct functions: putative cellular and developmental roles in insects, and a role in host defense in mammals [34]. However, *Drosophila* Toll-9 is structurally related to TLRs, both in its ectodomain and in the TIR domain as it is the only *Drosophila* Toll receptor that does not have N-flanking cystein-rich motifs at the C-terminal end of the ectodomain [35], [36]. Additionally, Toll-9 is, with Toll and to a less extend Toll-5, the only *Drosophila* Toll able to activate immune genes transcription upon over-expression experiments in tissue culture cells [37].

Finally, Toll-9 uses, at least in part, the same intracellular signaling components that Toll-1 to promote *Drosomycin* expression *in vitro* (i.e. DmMyD88 and Pelle [37]). All together, these data suggest that Toll-9 could be another Toll family members involved in *Drosophila* immunity. In order to test the putative implication of Toll-9 in the *Drosophila* immune response, we generated a *Toll-9* complete loss-of-function allele and tested its ability to mount an immune response. The following data suggest that Toll-9 is not required to mount an efficient response to bacterial infection.

## Materials and Methods

### Fly stocks

y,w; P{ry^+^, eyeless-FLP}6, ry^506^ (#5577); y,w; P{hsp70-FLP}11, P{hsp70-I-SceI}2B, noc^Sco^/CyO (#6934) and w; relish^E20^ e^s^ (#9457); hs-Gal4 (#2077); Df(3L)rdgC-co2, th^1^st^1^in^1^ kni^ri-1^p^p^/TM6C, cu^1^Sb^1^Tb^1^ca^1^ (#2052, deficiency that covers Toll-9, named Def(Toll-9)/TM6C in the text) were obtained from the Bloomington Drosophila Stock Center (http://flystocks.bio.indiana.edu/). spz^rm7^ allele has been described previously [12]. UAS-Toll-9 has been kindly provided by Tony Ip [38]. Serpent-Gal4, UAS-mCD8GFP and lozenge-Gal4, UAS-nlsGFP were kindly provided by Michele Crozatier. The Drosomycin-GFP and Cecropin-GFP lines were a gift from Bruno Lemaitre [39] and Won-Jae Lee [40] respectively. Escargot-Gal4, UAS-mCD8GFP and Diptericin-Cherry has been described [41], [42]. Axenic stocks were either obtained and maintained as described in [43], or obtained by growing flies on medium supplemented with antibiotics (100 µL of antibiotics cocktail per vial: Erythromycin, Kanamycin, Tetracyclin and Chloramphenicol, each at 10 mg/mL final). Adult flies were then kept at 25°C on autoclaved medium. The absence of bacteria in axenic stocks was verified by culturing homogenates of these flies on LB plates. For all other experiments, flies were kept on a standard cornmeal food at 25°C.

### Knock-out of the Toll-9 locus

**Figure 1:**
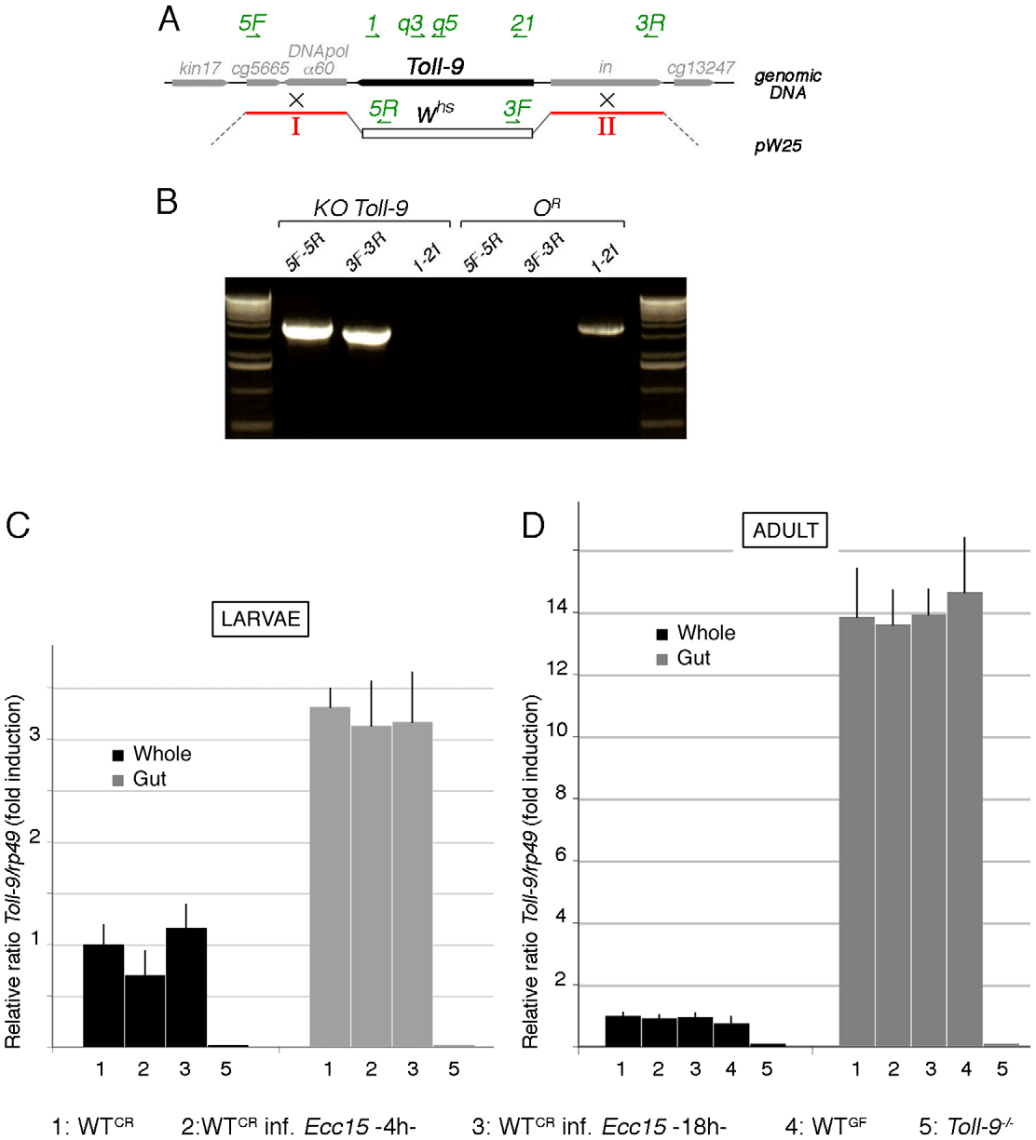
Knock-out of the *Toll-9* locus and *Toll-9* expression. (A) A complete deletion of the *Toll-9* locus was generated by targeted homologous recombination using 5′ (red, II) and 3′ (red, I) homology regions; the *white* cDNA replaced the *Toll-9* locus (black box in genomic region). (B) PCR performed on the resulting KO *Toll-9* line and Oregon^R^ control. The primers used are localized on the genomic region (A, green). (C) *Toll-9* expression is independent of commensal and infectious bacteria. *Toll-9* expression measured by quantitative RT-PCR in conventionally reared wild-type (-1- WT^CR^), conventionally reared wild-type 4 hours after *Erwinia carotovora carotovora 15* ingestion (-2- WT^CR^ inf. *Ecc15* -4 h-), conventionally reared wild-type 18 hours after *Ecc15* ingestion (-3- WTCR inf. *Ecc15* -16 h) and germ-free wild-type (-4- WT^GF^), in whole larvae, larval gut, whole adult and adult gut. *Toll-9* is enriched in the gut of both larvae and adult, and its expression level is not dependent on commensal and infectious bacteria. Additionally, *Toll-9* is no longer expressed in *Toll-9^−/−^* mutant (-5-). *rp49* was used as the experimental expression standard.

Knock-out of the *Toll-9* locus has been realized by ends-out gene targeting as described in [44]. The resulting deletion covers a genomic region starting from 852 bp upstream of the *Toll-9* ATG, and up to 41 bp after *Toll-9* stop codon. The 3′ and 5′ homology regions I (2926 bp long) and II (3116 bp long), showed in red in Figure 1, were amplified by PCR from *Oregon^R^* genomic DNA. The region I has been amplified using the following primers: F-ACATGCATGCATGTGGAAGCACTCTCGATTCAGC, R-GGGGTACCCCAGAGTTCTAGTCAGTTGTGC and contains *SphI* and *KpnI* sites respectively, whereas the region II has been amplified using the following primers: F-AGGCGCGCCTTCATCGGATACCCATTGAGG; R-CCCGTACGGGCGAGGATTCCGATAGATGCC and contains *AscI* and *BsiWI* sites. These two DNA fragments were then cloned into the pW25 vector (Drosophila Genomics Ressource Center), on both sides of the *miniwhite* gene *w^hs^*. This pW25-*Toll-9-*KO construct was then transformed into *y,w* embryos using the standard procedure for P-element [45]. Male donor flies were crossed with *y,w; hsp70-FLP, hsp70-I-SceI, noc^Sco^/CyO* females and the resulting progeny were heat-shocked at 37°C for one hour twice a day, at day 2 and 3 after egg laying. Mosaic and white eyes females were then collected from the progeny and crossed to males carrying an *eyeless-FLP* transgene on X chromosome. In the resulting progeny red eye males were screened. Out of 22.400 males, 26 red eye males were isolated but 7 of them were sterile. Genomic deletion of the *Toll-9* locus and replacement with the *w^hs^* gene were investigated by PCR, using 5F (5′-GCTGTTGACGAAGAGGGAAG-3′), 5R (GAATTGAATTGACGCTCCGT-3′), 3F (5′-GTCCGGTTGTTTTCGTGCTC-3′), 3R (5′-GTACACTTCCTTGGCTGGCG-3′), 1 (5′-CGTATTAGTATGCCTGTTCC-3′) and 21 (5′-ACAACTGACTAGAACTCTCC-3′) primers as indicated in Figure 1. Among the 19 lines established, only one showed ampification using the primer couples 5F–5R and 3F–3R, but no amplification using the primer couple 1–21 (Figure 1). This line has been isogenized using *y,w* flies, and kept as heterozygous in the same vial. Consequently, the control *y,w* has white eyes, the heterozygous *y,w; Toll-9^−^/+* has orange eyes, and the *y,w; Toll-9^−^/Toll-9^−^* mutant has red eyes.

### Hemocytes count

For each genotype *(Toll-9^−^/TM3_GMR_YFP* and *Toll9^−/−^* larvae), 4 wandering third instar larvae were bled onto a coverslip, and the hemocytes collected were incubated for 10 minutes in a humidified chamber to allow them to adhere to the slide, and then fixed in PFA 4% for 5 minutes. The cells were thereafter rinsed 3 times in PBS and mount in Vectashield with DAPI. The results are the mean of 4 different experiments, and each experiment is the mean of 3 counts.

### Longevity assays

Assays for longevity were performed on normal food or on food supplemented with an antibiotics cocktail. For each genotype (*y,w, Toll-9^−/−^* and *Toll-9^−^/Def(Toll-9)*) at least 80 flies grouped by 10 males in independent vials were tested.

### Immunocytochemistry

Immunocytochemistry was performed as previously described (McKearin and Ohlstein, 1995). The mouse anti-Delta (1∶20, Developemental Studies Hybridoma Bank), and chicken anti-GFP (1∶500, Aves Labs) were used in this study. Fluorescence-conjugated secondary antibodies were purchased from Interchim Jackson (Alexa546 goat anti-mouse IgG antibody) and Molecular probes (Alexa488 goat anti-chicken antibody), and used at a 1∶500 dilution. For DNA labeling, tissues were incubated with 1 µg/mL of DAPI (Sigma). All samples were mounted in Vectashield mounting medium (Vectorlabs) and examined using an apotome microscope (Zeiss Axioplan 2). Images were acquired using AxioImager and composite figures were prepared using Adobe Photoshop CS3.

### Quantitative Real-Time PCR

To quantify the gene expression, fluorescence real-time PCR was permormed using the double-stranded DNA-dye SYBR Green (From Invitrogen). Primer pairs for *Toll-9* (sense, q5, 5′-CCACTCTGGAAATGGCCTTA-3′; antisense, q3, 5′-GGTAAATCAGTTCCGGCAGA-3′), *Diptericin* (sense, 5′-GCTGCGCAATCGCTTCTACT-3′; antisense, 5′-TGGTGGAGTGGGCTTCATG-3′), *Drosomycin* (sense, 5′-CGTGAGAACCTTTTCCAATATGATG-3′; antisense, 5′-TCCCAGGACCACCAGCAT-3′), *Defensin* (sense, 5′-GTTCTTCGTTCTCGTGG-3′; antisense, 5′-CTTTGAACCCCTTGGC-3′), *Drosomycin3* (sense, 5′-CAGATGATATTCCTGTTTGCT-3′; antisense, 5′-TGTCCCTCCTCAATGC-3′), *rp49* (sense, 5′-GACGCTTCAAGGGACAGTATCTG-3′; antisense, 5′-AAACGCGGTTCTGCATGAG-3′), were used to target gene transcripts. All samples were analyzed in triplicates, and the levels of detected RNA were normalized using *rp49* as a control.

### Bacterial strains and infection experiments

Bacterial strains used were Escherichia coli, Erwinia carotovora carotovora 15, Pseudomonas entomophila, Lactobacillus plantarum, Serratia marcescens and Enterococcus faecalis. Systemic bacterial infections were performed by septic injury, consisting of wounding the adult thoracic cuticle with a needle previously dipped into a concentrated bacterial solution (OD = 200 for E. coli and Ecc15, and OD = 20 for E. faecalis). Larval oral infections were performed by adding 200 µL of concentrated bacteria solution (OD = 200) directly on L2-L3 larvae. Adult oral infections were performed by letting flies feed on a 2.5% sucrose solution contaminated with concentrated bacteria (final OD = 100).

## Results

### Phenotypic characterization of *Toll-9* mutant

**Figure 2:**
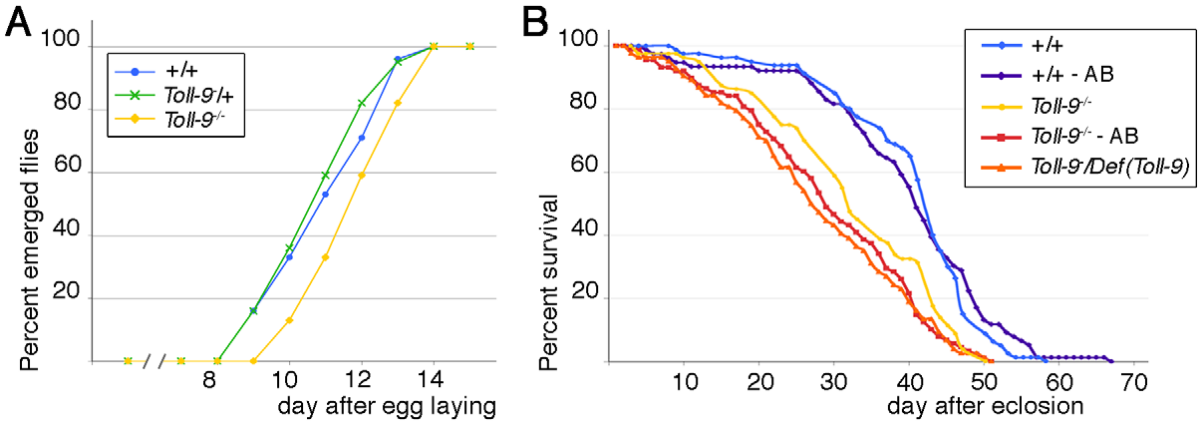
*Toll-9^−/−^* mutant emerging length is delay and lifespan is reduced. (A) *Toll-9^−/−^* mutant emergence is delayed of one day compared to wild-type +/+ and heterozygous *Toll-9^−^*/+. (B) *Toll-9^−/−^* and *Toll-9^−^/Def(Toll-9)* mutant lifespan is reduced. Median survival is of 32 and 27 days respectively for mutants and 42 days in controls. This reduction is still observed if flies grown on food supplemented with antibiotics (29 in *Toll-9^−/−^* versus 41 days in controls).

In order to study Toll-9 receptor function, we generated a complete loss-of-function allele by targeted homologous recombination [44]. 5′ and 3′ flanking regions of homology were introduced into the pW25 vector (Figure 1A) to generate transgenic flies. The *Toll-9* gene disruption knock-out candidate flies were screened and verified using PCR (with specific primers in the two flanking regions of *Toll-9* -5F and 3R-, within *Toll-*9 -1 and 21- and within *w^hs^* -5R and 3F-, Figure 1A, B) and quantitative PCR (using q5 and q3 primers, Figure 1C, D). *Toll-9^−/−^* mutants in which both maternal and zygotic contributions have been removed hatched into morphologically normal and fertile adults, although the *Toll-9^−/−^* flies emerged one day later than the *w* control. Indeed, the t_50_ (time by which 50% of the progeny has emerged) is of 10.7 days for the control and of 11.6 days for *Toll-9^−/−^* (Figure 2A). Moreover, *Toll-9^−/−^* and *Toll-9^−^/Def(Toll-9)* flies showed a reduced lifespan (Figure 2B) with the mean lifespan of *w* control flies being at 42 days versus 32 and 27 days for *Toll-9^−/−^* and *Toll-9^−^/Def(Toll-9)* mutant flies, respectively. A similar lifespan shift was observed when flies are grown in axenic conditions (Figure 2B). Finally, an embryonic lethality was observed, since only one third of the expected *Toll-9^−/−^* embryos hatched into larvae. The analysis of the dead embryos revealed that the lethality occurs at the end of embryogenesis. In conclusion, loss of *Toll-9^−/−^* function is associated with partial embryonic lethality, delay in adult flies emergence and reduced lifespan.

**Figure 3:**
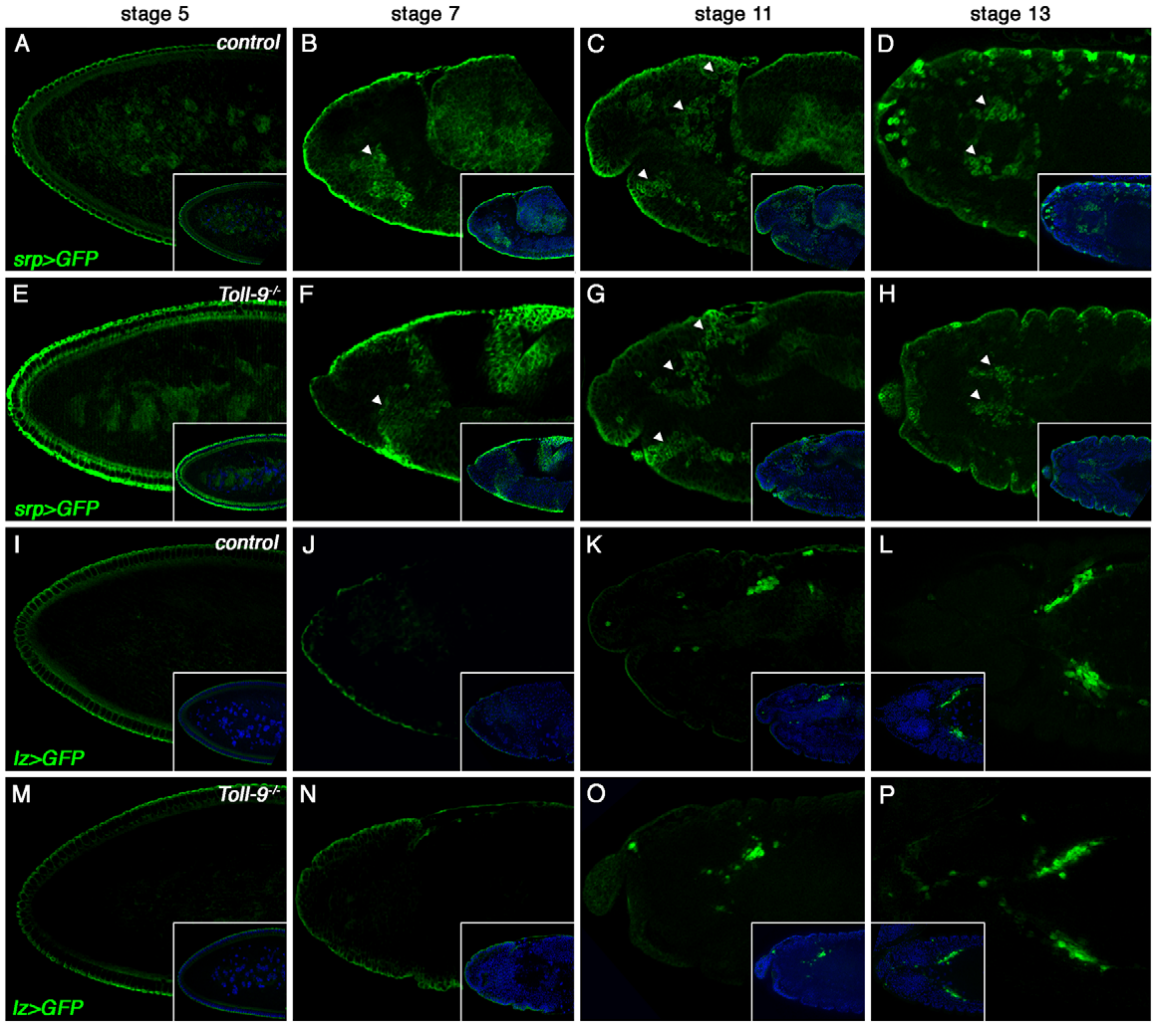
Hematopoiesis occurs normally in *Toll-9^−/−^* mutant embryos. *Serpent-GFP* and *Lozenge-GFP* expression in stages 5, 7, 11 (side views) and 13 (dorsal views) of control (A, B, C, D, *Srp-GFP*; and I, J, K, L, *Lz-GFP*, respectively) and *Toll-9^−/−^* (E, F, G, H, *Srp-GFP*; and M, N, O, P, *Lz-GFP*, respectively) embryos. The *Srp* transcription factor is expressed in hemocytes precursors, which will differentiate into plasmatocytes and crystal cells. The AML-1/Runx1 homolog *Lz* is required for crystal cell differentiation and expressed in a subset of prohemocytes. During blastoderm stage, *Srp* is expressed in the yolk nuclei (A). After gastrulation and thenafter, *Srp* expression is found in the putative hemocyte primordium within the anterior mesoderm primordium (B, C, D, arrowheads), and in the primordium of the posterior midgut (B, C, arrows). *Lz* is first detected in stage 10, in a small cluster of cells within the head mesoderm (K, L), corresponding to the crystal cells precursors, a subset of Srp-expressing hemocyte precursors. The *Srp*- and *Lz*- expressing cells in *Toll-9^−/−^* mutant embryos are numerically, morphologically and spatially unchanged compared to wild-type.

*Toll-9* expression is highly spatially and temporally restricted during embryogenesis [46]. It starts at stage 5 in the vitellophages and is, from stages 7 to 11, limited to a group of cells in the head region, located at the site where the hemocytes progenitors are specified. Later in embryogenesis, *Toll-9* transcripts are no longer detectable [46]. Knowing that hemocytes are essential for normal embryogenesis to take place and are playing essential roles in immunity [42], [47], [48], we checked whether the embryonic hemocyte population was affected in *Toll-9^−/−^* mutants. For that purpose, we monitored the expression of two hematopoietic markers, Serpent, a GATA transcription factor expressed in hemocytes precursors which will differentiate into plasmatocytes and crystal cells, and Lozenge, an AML-1/Runx1 homologue required for crystal cell differentiation and expressed in a subset of prohemocytes [49], [50]. As showed in Figure 3, both factors were expressed normally in *Toll-9^−/−^* mutant embryos (even at the end of embryogenesis, data not shown), suggesting that the embryonic hematopoiesis is not affected by the absence of Toll-9. This was further confirmed by counting the number of larval hemocytes. We found that the number of larval hemocytes was slightly increased in *Toll9^−/−^* mutants. Indeed, we found 1775±238 hemocytes per mm^2^ (n = 11420) in *Toll-9^−/−^* mutant larvae, compared to 1242±138 hemocytes per mm^2^ (n = 7990) in *Toll-9^−^/TM3_GMR_YFP* control larvae (less than a 2-fold increase). It is however unlikely that this increase of embryonic hemocytes could explain the partial embryonic lethality observed in *Toll-9^−/−^* stocks.

### Toll-9 function is not required for basal and inducible AMP production

**Figure 4:**
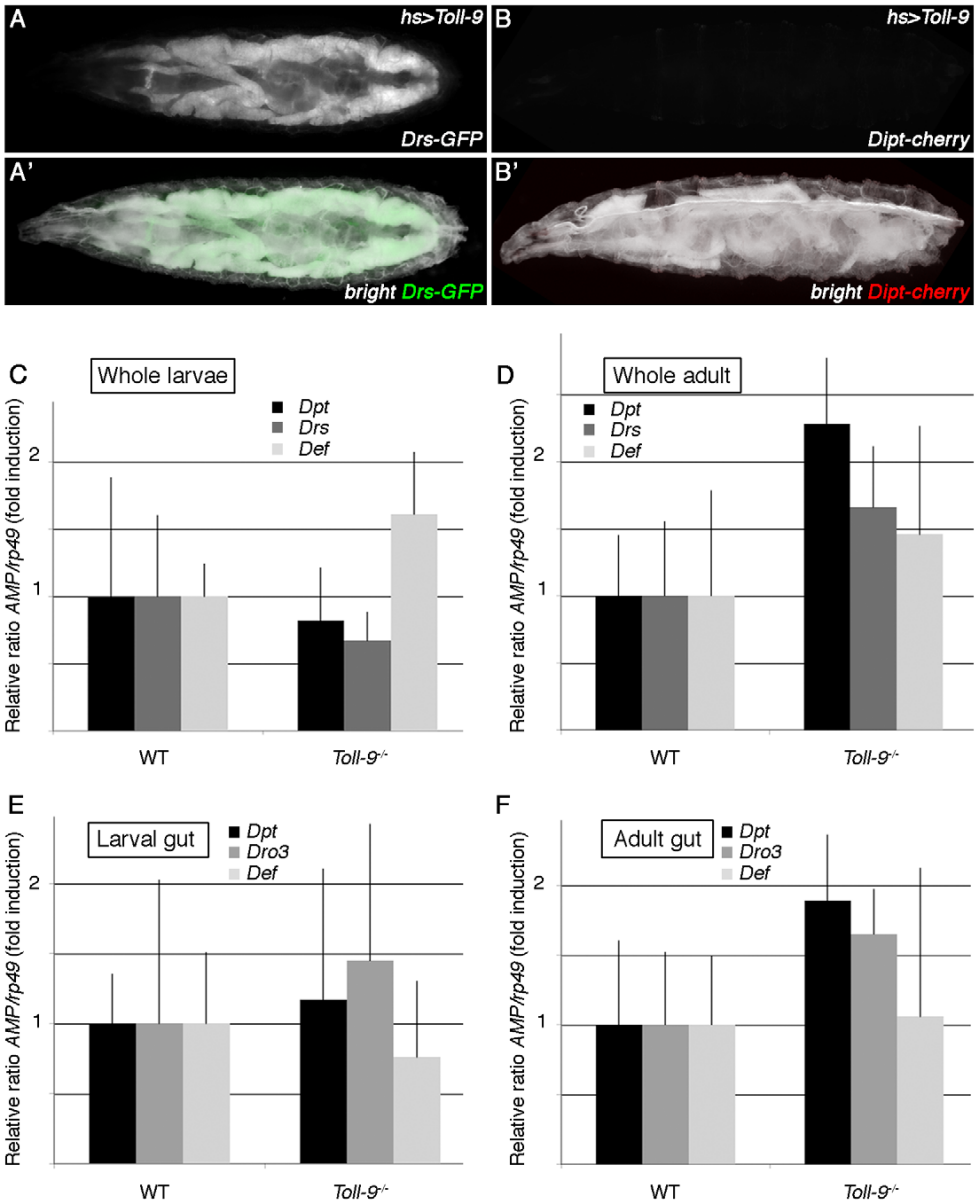
Basal AMP mRNA levels are identical in wild-type and *Toll-9^−/−^* mutants. (A–B) AMPs expression in larvae overexpressing *Toll-9*. *Drs-GFP* (A, A') and *Dpt-Cherry* (B, B') expression in *Drs-GFP; hs-GAL4/UAS-Toll9* and *hs-GAL4/UAS-Toll9; Dpt-Cherry* larvae respectively, after a heat-shock treatment. Overexpressing *Toll-9* in the larva using a constitutive driver leads to the ectopic expression of *Drosomycin*, but not *Diptericin*. (C–F) Quantitative RT-PCR analysis of AMPs production in unchallenged *Toll-9^−/−^* mutants whole larvae (C), whole adult (D), larval gut (E) and adult gut (F) compared to control. *Diptericin* (C–F), *Drosomycin* (C, D), *Defensin* (C–F) and *Drosomycin3* (E, F) expression were not significantly different in a *Toll-9^−/−^* mutant background compared to controls (for each experiment, P≥0.06). Relative *AMP/rp49* ratios of controls were set to 1 to indicate fold induction.

It has been shown that Toll-9 overexpression *in vitro* and *in vivo* is sufficient to induce the expression of antimicrobial peptides genes known to be regulated by the Toll pathway, such as *Drosomycin* or *Cecropin* ([37], [38] and Figure 4A, A'), but not *Diptericin*, an Imd pathway regulated gene (Figure 4B, B'). This work had led to the idea that Toll-9 functions as a constitutively active signaling receptor that will maintain a substantial level of antimicrobial peptides required to ward off continuous challenge of microorganisms.

We then wanted to test whether *Toll-9* expression was dependent on the presence of either commensal or pathogenic bacteria. We therefore compared Toll-9 transcript levels between flies grown in axenic conditions, conventionally-reared flies, and flies infected with the phytopathogenic bacteria *Erwinia carotovora carotovora 15* (*Ecc15*). Our results indicate that *Toll-9* expression was neither modified under axenic conditions nor after immune challenge by *Ecc15* (Figure 1C, D). This is in agreement with previously published results showing that Toll-9 transcript levels did not seem to change in the whole larvae and adult following septic injury with *Escherichia coli* [37].

Among immune-competent tissues, the gut is permanently in contact with a large number of well-tolerated commensal bacteria, which under normal condition do not elicit an immune response. Our results, together with microarray analysis (http://www.flyatlas.org), indicate that Toll-9 is highly and specifically enriched in larval and adult guts (3.3 times in the larval midgut compared to whole larvae, Figure 1C and 13.8 times in the adult midgut, compared to whole fly, Figure 1D). It was therefore tempting to speculate that Toll-9 may play a role in localized epithelial defense by maintaining a basal level of antimicrobial peptides in the gut, a tissue in constant contact with microorganisms. We then tested whether Toll-9 expression was modified specifically in the gut in axenic conditions or following oral infection with *Ecc15*. Contrary to *Bombyx mori Toll-9,* whose expression is significantly increased in the gut by microbial challenge [51], *Drosophila Toll-9* expression levels was not significantly different in conventionally reared flies, flies grown in axenic conditions or in immune challenged flies (Figure 1C, D).

**Figure 5:**
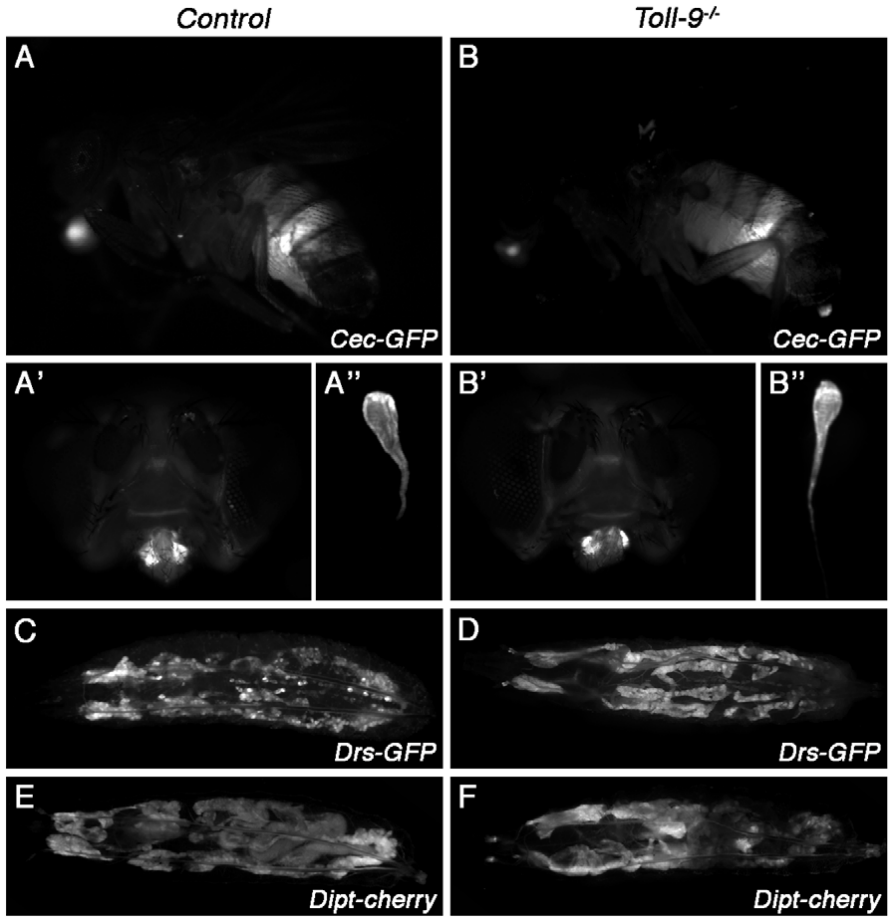
Epithelial AMP expression pattern is identical in wild-type and *Toll-9^−/−^* mutants. (A–B) In unchallenged adult flies (A, B), *Cecropin-GFP* constitutive expression observed in the labellar glands (A', B') and in the dissected spermathecae (A” and B”) is unchanged in the *Toll-9^−/−^* mutant. 18 hours after *Ecc15* ingestion, *Drosomycin-GFP* (C, D) and *Diptericin-Cherry* (E, F) are similarly expressed in the fat body of *Toll-9^−/−^* mutant and control larvae.

To further test the hypothesis that Toll-9 is required to maintain a basal level of antimicrobial peptides, we measured the levels of several peptides in *Toll-9^−/−^* mutants, in the whole animal to quantify the systemic response, and specifically in the gut to measure the local response. The following antimicrobial peptides were tested: *Drosomycin* and *Defensin*, regulated by the Toll pathway, *Diptericin*, regulated by the Imd pathway, and *Drosomycin3*, a gut expressed gene whose transcription is JAK/STAT pathway dependent [52]. *Toll-9^−/−^* mutant larvae and adults were always compared to *Toll-9^−^/TM3* control larvae and +/+ control adults, respectively, from the same vial, to avoid variations due to external factors. Basal expression levels of *Diptericin*, *Drosomycin*, *Defensin* (Figure 4C, D) in whole animals and of *Diptericin*, *Drosomycin3* and *Defensin* (Figure 4E, F) in guts were not significantly different in controls and *Toll-9^−/−^* mutants (for each experiment, P≥0.06). We confirmed this result using antimicrobial peptide reporter lines. Indeed, uninfected wild-type and *Toll-9^−/−^* mutants show identical *Diptericin-Cherry*, *Drosomycin-GFP* and *Cecropin-GFP* expression pattern in both larvae and adult (constitutive *Cecropin-GFP* expression in the adult is shown in Figure 5A, B).

**Figure 6:**
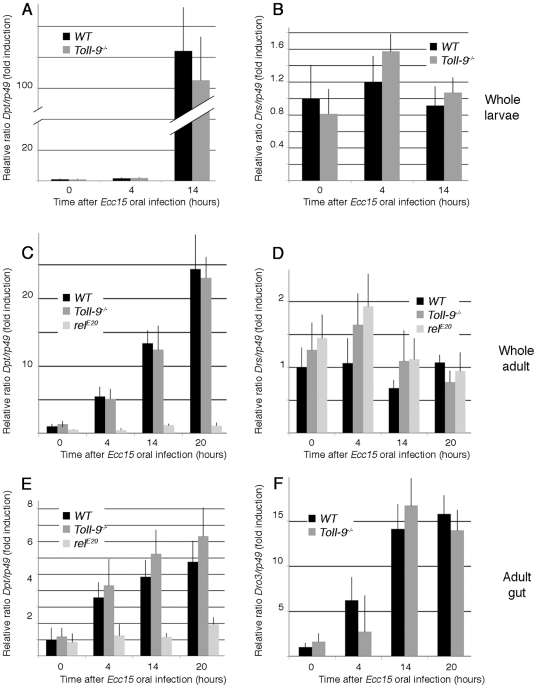
Systemic AMP production is independent of Toll-9 function. Quantitative RT-PCR analysis of AMPs induction after Gram-negative bacteria ingestion. *Diptericin* (A. C, E), *Drosomycin* (B, D) and *Drosomycin3* (F) expression upon *Ecc15* oral ingestion in *Toll-9^−/−^* whole larvae (A, B), whole adults (C, D), and adult gut (E, F), and in *relish^E20^* whole adult (C, D), and adult guts (E), compared to control. *rp49* was used as the experimental expression standard. Relative *AMP/rp49* ratios of unchallenged controls were set to 1 to indicate fold-induction.

The data above suggest that contrary to what has been proposed earlier, Toll-9 is not required to maintain a basal level of AMPs production *in vivo*. We then tested whether Toll-9 is necessary to regulate immune gene induction after microbial challenge. As *Toll-9* is mainly expressed in the gut, we used the *Ecc15* oral infection model. First, we observed that the survival of *Toll-9^−/−^* mutants was not affected by *Ecc15* oral infection (Figure S1). We next checked induction levels of *Diptericin* and *Drosomycin* in whole larvae and adult, and *Diptericin* and *Drosomycin3* expression in the adult gut (Figure 6). Since antimicrobial peptides gut induction has been shown to depend essentially on the Imd pathway, we used *relish^E20^* as a control. Upon *Ecc15* oral infection, *Diptericin* expression increased up to 120-fold in whole larvae (Figure 6A), 25-fold in whole adults (Figure 6C) and 6 times in adult guts (Figure 6E). Similar induction levels were obtained in *Toll-9^−/−^* mutant background (Figure 6A, C, E, for each experiment, P≥0.25), whereas, as expected, *Diptericin* induction was abolished in a *relish^E20^* mutant (Figure 6C, E). Similarly, no significant variation was observed for *Drosomycin* and *Drosomycin3* induction between wild-type and *Toll-9^−/−^* mutants (Figure 6B, D, F). These results were confirmed by using reporter lines for *Cecropin*, *Drosomycin* and *Diptericin* (*Cec-GFP*, *Drs-GFP* and *Dpt-cherry*, respectively), both in the larvae (Figure 5C–F) and in the adult (data not shown). To complete this analysis, we performed oral infection using other bacteria species such as *Pseudomonas entomophila*, *Serratia marcescens* and *Lactobacillus plantarum*. Twenty hours following oral ingestion of these bacteria, *Diptericin* mRNAs induction was not significantly different in *Toll9^−/−^* versus control midguts (Figure S2, for each experiment, P≥0.1). From these experiments we concluded that the basal and oral infection-induced antimicrobial gene levels are unaffected by *Toll-9^−/−^* inactivation. This suggests that Toll-9 does not participate in maintaining basal expression levels of antimicrobial peptides genes in normal conditions or after oral immune challenge.

**Figure 7:**
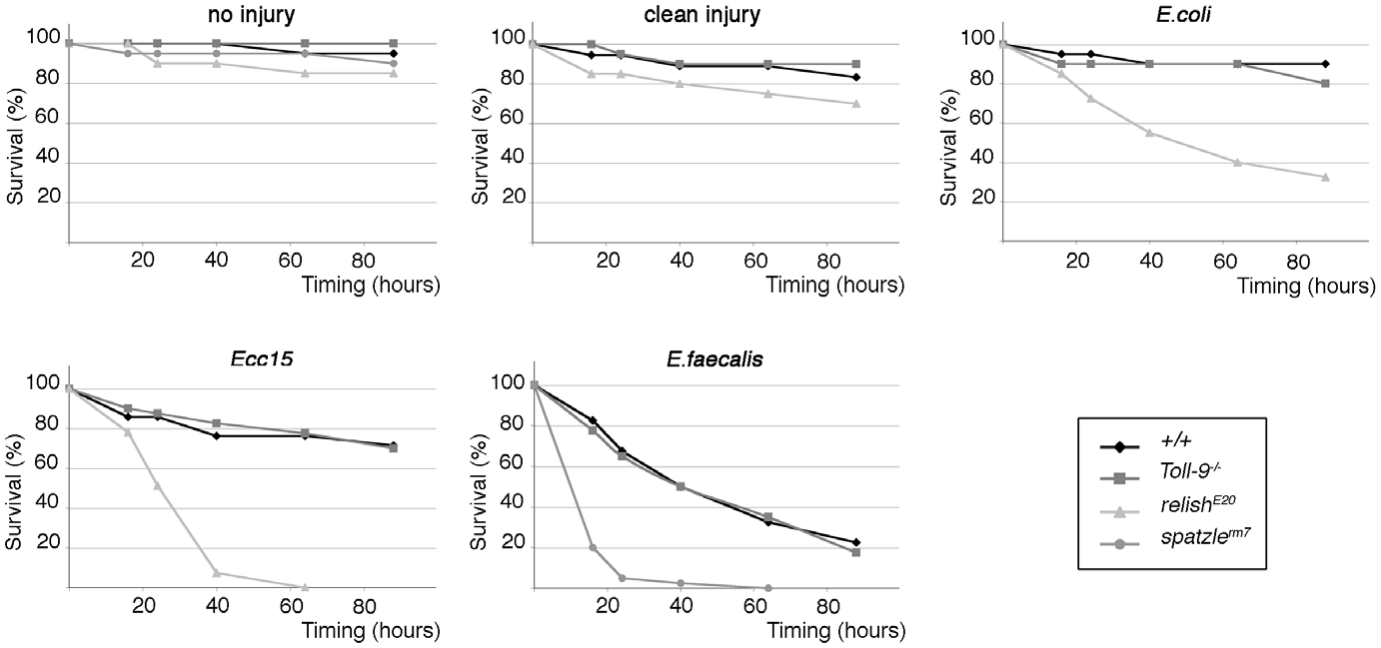
Resistance to bacterial infection is not impaired in *Toll9^−/−^* adults. Survival curves of *Toll-9^−/−^*, *relish^E20^* and *spz^rm7^,* and control flies in absence of injury, upon clean injury or after septic injury with *E. coli*, *Ecc15* or *E. faecalis*.

To complete these experiments, we analyzed AMP production and survival of *Toll-9^−/−^* flies infected by septic injury. Our results showed that *Toll-9^−/−^* mutant flies resist as well as control flies to clean injury and to septic injuries performed with either Gram-positive or Gram-negative bacteria (Figure 7). We next wanted to test whether the humoral antimicrobial response was affected in *Toll-9^−/−^* infected mutant. *Diptericin* mRNA induction upon *Ecc15* infection was similar in *Toll-9^−/−^* mutant and in controls (Figure S3), indicating that Imd dependent AMP production response was not affected by the absence of Toll-9.

### Gut homeostasis is normal in *Toll-9* mutant

**Figure 8:**
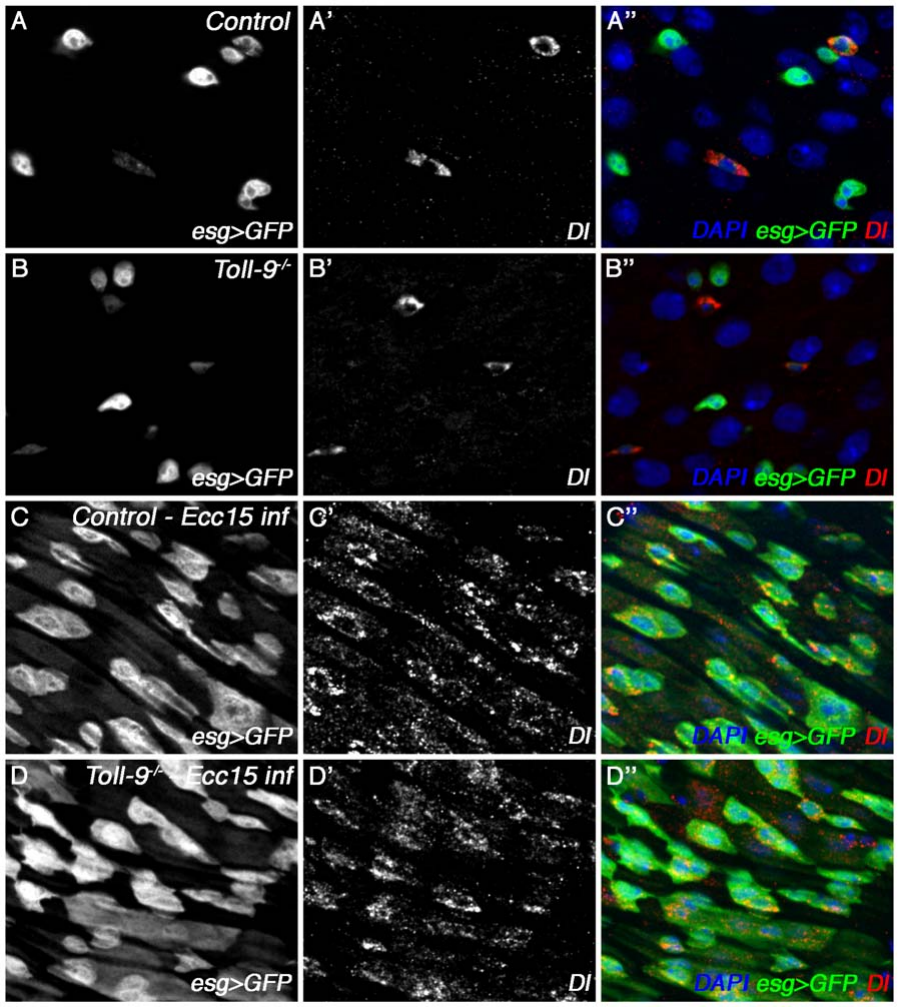
Gut epithelium renewal is not affected in *Toll-9^−/−^* mutants. Immunostaining with antibodies against GFP (white, A–D; green, A”–D”) and Delta (white, A'–D'; red, A” –D”). DNA is shown in blue (A”–D”). *Esg*-GFP positive cells correspond to ISCs or young enteroblasts, whereas Dl stains only ISCs in unchallenged adult guts (A, B). 20 hours after *Ecc15* oral infection, Esg-GFP positive cell and Dl positive cell numbers strongly increase (C–D), corresponding to an increased epithelium renewal, observed both in the control and in *Toll-9^−/−^* guts.

Recent studies have revealed that bacterial infection can trigger a gut immune response that involves the production of Reactive Oxygen Species and antimicrobial peptides [52]–[55]. Associated with this mechanism of defense, the gut epithelium triggers the proliferation and differentiation of intestinal stem cells (ISCs, stained with Delta (Dl) and Escargot (Esg), [52]. Upon stimulation, the ISCs give rise to two intestinal cell types: enterocytes (young enterocytes still express Esg) and enteroendocrine cells [56]. It has been shown that oral infection with some bacteria species can stimulate epithelium renewal by inducing ISCs proliferation and therefore production of enterocytes [52], [53]. We then tested whether the gut homeostasis was modified in a *Toll-9^−/−^* mutant. As shown in Figure 8, the numbers of ISCs (Esg-positive and Dl-positive cells) and young enterocytes (Esg-positive and Dl-negative cells) were similar in unaffected *Toll-9^−/−^* mutants and in control guts (Figure 8A–A” and B–B”). Upon infection, the epithelium renewal occurred normally in a *Toll-9^−/−^* mutant midguts (Figure 8C–C” and D–D”). These results indicate that neither the basal gut renewal in non-infected guts nor the epithelium renewal triggered by bacterial infection is dependent on Toll-9 function.

## Discussion

The findings that TLRs are implicated in the immune response in mammals and that Toll participates in the *Drosophila* host response has led to the idea that TLR-mediated immunity is originating from ancestor of bilaterian (review in [18]). If such, one might expect multiple mammalian TLRs and *Drosophila* Toll family members to have immune function. If it is now clearly demonstrated that every single mammalian TLR family member is implicated in an immune mechanism, it is however not the case for *Drosophila* for which evidence for immune role has only been gathered for the Toll-1 protein itself [12], [57], [58]. In contrary, there are some reasons to think that the immune implication of TLRs and Toll have been co-opted independently in insects and mammals [36], [59], [60]. This hypothesis is based on functional differences between Toll and TLR implication in immunity, the most obvious being the difference in their mode of activation. Whereas TLR receptors are directly activated by versatile microbial ligands, *Drosophila* Toll-1 receptor activation is dependent of the maturation of its Spätzle ligand by proteolytic cleavage [18], [61], [62]. The goal of this study was to test whether another member of the Toll family other than Toll-1 itself could have an immune function in flies. We focused our attention on Toll-9 for two reasons. 1) Its extracellular domain organization resembles that of the TLRs [35], and 2) published gain-of-function experiments have previously suggested that Toll-9 could function as a constitutively active receptor triggering basal AMP production [37]. However, the phenotypic characterization of a complete loss-of-function mutant presented here tends to indicate that Toll-9 is not implicated in the antibacterial arm of the immune response. Indeed, Toll-9 is neither required for basal and immune inducible AMP production nor for intestinal response to oral bacterial infection. In addition, our results rules out the hypothesis that Toll-9 is triggering a constant baseline level of AMPs required to eliminate pathogens when they enter the body cavity. We can however not exclude that a functional Toll-9 receptor is not of importance to mediate other aspects of the immune response such as anti-viral or and-parasitic immunities. It is of note that the Toll signaling pathway control anti-dengue virus in *Aedes aegypti* [63]. It is also possible that Toll-9 is functionally redundant with other Toll proteins, although the rather unique extracellular domain organization of Toll-9 let us believe that this is unlikely. What is then the function of Toll-9? We have identified an incompletely penetrant embryonic lethality associated with the loss of Toll-9. It is however difficult to pinpoint the exact cause of this lethality. The only clear phenotype associated with *Toll-9* elimination is an increase of blood cell number. It is however well know that the number of circulating hemocytes is highly dependent on genetic background and can strongly fluctuate even in between wild type stocks. We therefore do not favor the hypothesis than the high hemocytes figures in *Toll-9^−/−^* mutants are responsible for embryonic lethality. Further work will be needed to dissect the precise role of Toll-9 during embryogenesis. It should however be noted that both TLRs and *Drosophila* Toll-1 have been shown to be important for non-immune function. Whereas Toll-1 is also involved in early axis determination and in specifying neuromuscular innervation pattern, the number of manuscripts describing TLR expression and function in the central nervous system (CNS) has been increasing steadily and expanding beyond their traditional roles in infectious diseases to neurodegenerative disorders and injury [14], [64]–[71]. This might be a route to follow for finding Toll-9 function!

## Supporting Information

Figure S1 ***Toll-9^−/−^*** **mutant survival is not affected by** ***Ecc15*** **oral infection.** 7 days after oral ingestion of the *Ecc15* bacteria, *Toll-9^−/−^* mutant flies survive as well as the control.
(TIF)

Figure S2 **Gut** ***Diptericin*** **expression after bacteria feeding is not modified in** ***Toll-9^−/−^*** **mutant.** Quantitative RT-PCR analysis of *Diptericin* expression in *Toll-9^−/−^* mutant and control guts after oral challenge with *Ecc15*, *Lactobacillus plantarum*, *Pseudomonas entomophila* and *Serratia marcescens*. Relative *Diptericin/rp49* ratios of unchallenged controls were set to 1 to indicate fold induction.
(TIF)

Figure S3 ***Diptericin*** **mRNA levels after** ***Ecc15*** **septic injury are comparable in wild-type** ***Toll-9^−/−^*** **adults.** Quantitative RT-qPCR analysis of *Diptericin mRNA* 6 and 24 hours after septic injury with *Ecc15* in wild-type and *Toll-9^−/−^* mutant adults. *Diptericin/rp49* ratio at 6 hours post challenged was set as 100%.
(TIF)
