# Supplementary figures and images for: Lack of an Antibacterial Response Defect in Drosophila Toll-9 Mutant
Source: PLoS One. 2011 Feb 28;6(2):e17470. doi: 10.1371/journal.pone.0017470 (PMC3046252; doi:10.1371/journal.pone.0017470)

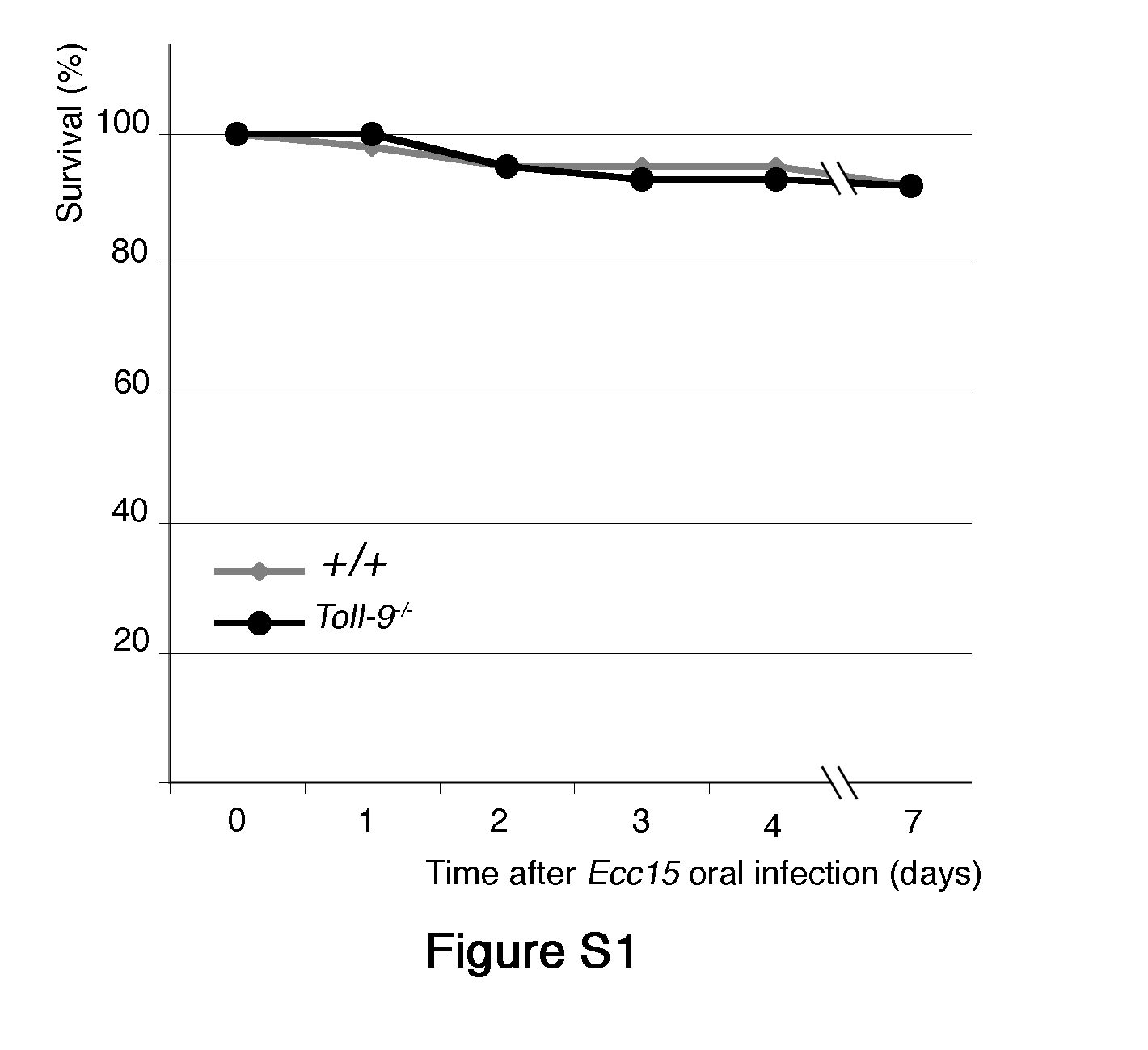

Supplement: Figure S1 — Toll-9−/− mutant survival is not affected by Ecc15 oral infection. 7 days after oral ingestion of the Ecc15 bacteria, Toll-9−/− mutant flies survive as well as the control. (TIF) [file pone.0017470.s001.tif]

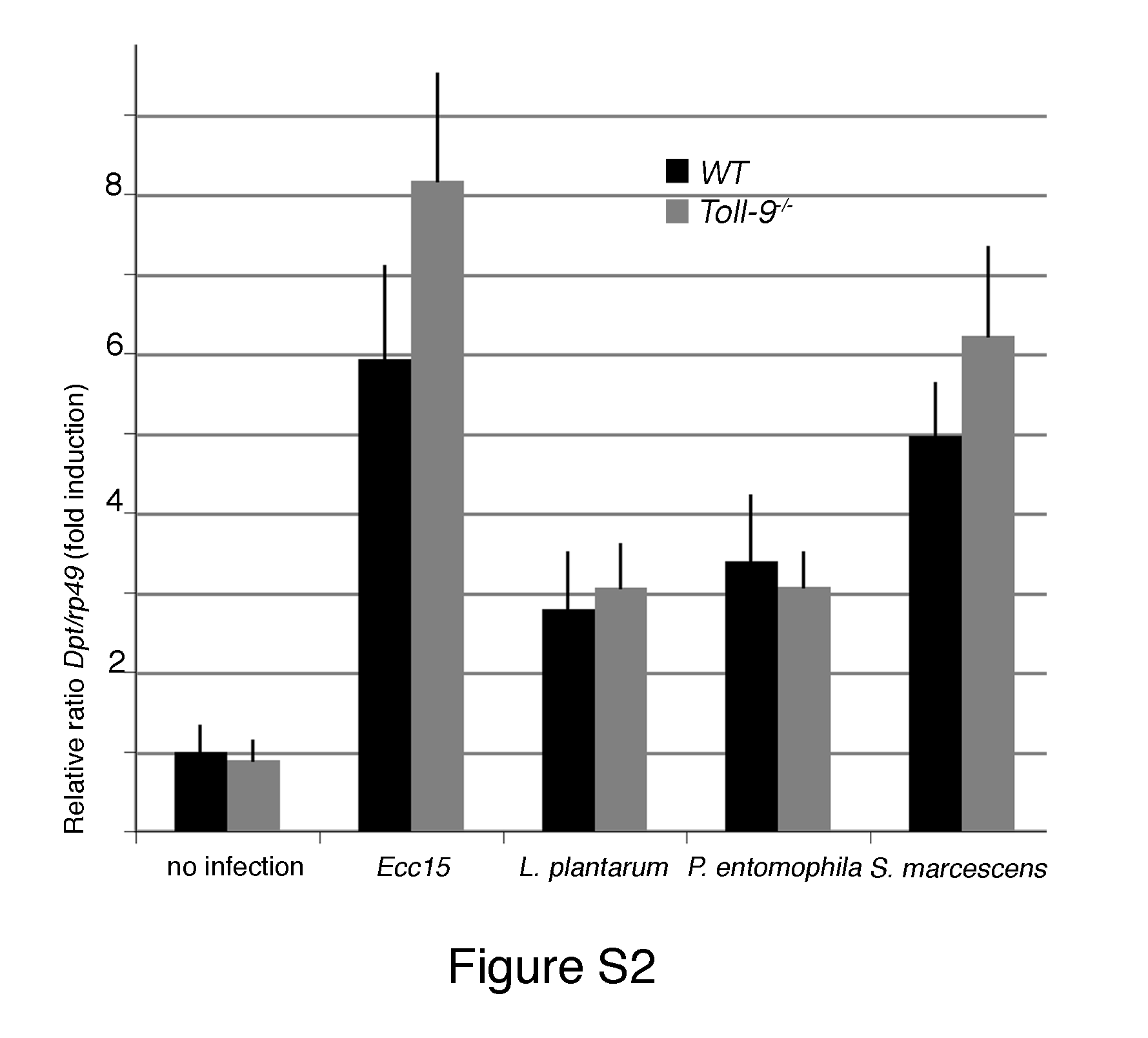

Supplement: Figure S2 — Gut Diptericin expression after bacteria feeding is not modified in Toll-9−/− mutant. Quantitative RT-PCR analysis of Diptericin expression in Toll-9−/− mutant and control guts after oral challenge with Ecc15, Lactobacillus plantarum, Pseudomonas entomophila and Serratia marcescens. Relative Diptericin/rp49 ratios of unchallenged controls were set to 1 to indicate fold induction. (TIF) [file pone.0017470.s002.tif]

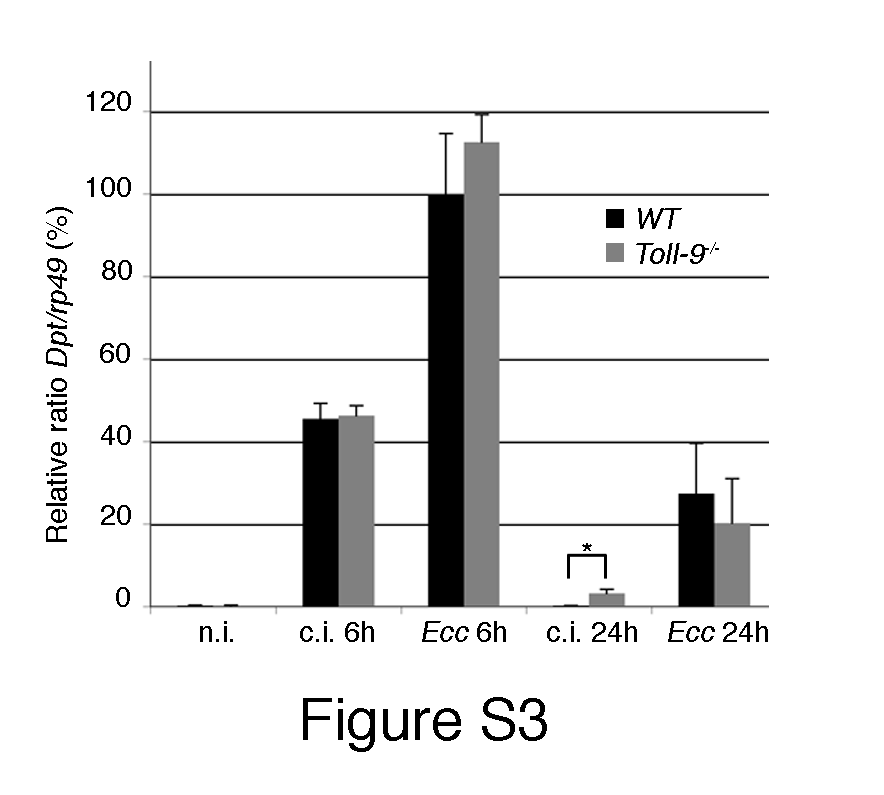

Supplement: Figure S3 — Diptericin mRNA levels after Ecc15 septic injury are comparable in wild-type Toll-9−/− adults. Quantitative RT-qPCR analysis of Diptericin mRNA 6 and 24 hours after septic injury with Ecc15 in wild-type and Toll-9−/− mutant adults. Diptericin/rp49 ratio at 6 hours post challenged was set as 100%. (TIF) [file pone.0017470.s003.tif]
